# Generalized transformations and coordinates for static spherically symmetric general relativity

**DOI:** 10.1098/rsos.171109

**Published:** 2018-04-11

**Authors:** James M. Hill, Joseph O'Leary

**Affiliations:** 1 School of Information Technology and Mathematical Sciences, University of South Australia, PO Box 2471, Adelaide, South Australia 5001, Australia; 2 SERC Limited, AITC2 Mount Stromlo Observatory, Canberra, Australian Capital Territory 2611, Australia

**Keywords:** exact solutions, Einstein field equations, coordinate transformation

## Abstract

We examine a static, spherically symmetric solution of the empty space field equations of general relativity with a non-orthogonal line element which gives rise to an opportunity that does not occur in the standard derivations of the Schwarzschild solution. In these derivations, convenient coordinate transformations and dynamical assumptions inevitably lead to the Schwarzschild solution. By relaxing these conditions, a new solution possibility arises and the resulting formalism embraces the Schwarzschild solution as a special case. The new solution avoids the coordinate singularity associated with the Schwarzschild solution and is achieved by obtaining a more suitable coordinate chart. The solution embodies two arbitrary constants, one of which can be identified as the Newtonian gravitational potential using the weak field limit. The additional arbitrary constant gives rise to a situation that allows for generalizations of the Eddington–Finkelstein transformation and the Kruskal–Szekeres coordinates.

## 1. Introduction

The first exact solution to the empty space field equations of general relativity is due to Karl Schwarzschild [1]. The derivation is now commonplace and can be readily found in the literature (e.g. [2–7]). It describes the space–time outside a spherically symmetric, static and asymptotically flat body of mass *M*.

The line element in Schwarzschild geometry in spherical coordinates *x*^*μ*^=(*ct*,*r*,*θ*,*φ*) is given by

1.1 $$ds^{2}=\left(1+\frac{r_{0}}{r}\right)dt^{2}-{\left(1+\frac{r_{0}}{r}\right)}^{-1}\;dr^{2}-r^{2}\;d\Omega^{2},$$

where $d\Omega^{2}=d\theta^{2}+\sin^{2}\theta \;d\varphi^{2}$, *r*_0_=−2*GM*/*c*^2^, and the speed of light in vacuum and Newton’s gravitational constant are given by *c* and *G*, respectively, and here we adopt a time scale in which *c*=1.

The standard approach in deriving the Schwarzschild solution is to consider one of the following spherically symmetric line elements of the form:

1.2 $$\begin{array}{r} ds^{2}=a(r)\;dt^{2}-c(r)\;dr^{2}-r^{2}\;d\Omega^{2}, \end{array}$$

1.3 $$\begin{array}{r} ds^{2}=a(r,t)\;dt^{2}-c(r,t)\;dr^{2}-r^{2}\;d\Omega^{2} \end{array}$$

1.4 $$\begin{array}{r} \;\mathrm{and}\;ds^{2}=a(r,t)\;dt^{2}+2b(r,t)\;dr\;dt-c(r,t)\;dr^{2}-r^{2}\;d\Omega^{2}, \end{array}$$

where *a*,*b* and *c* denote unknown functions of either space or both space and time which are to be determined. Throughout the literature, convenient coordinate transformations [8,9] involving the introduction of a new time coordinate, allow for the removal of the non-orthogonal component in equation (1.4) and physical arguments such as a static space–time [7] inevitably lead to the Schwarzschild solution.

The aforementioned assumptions then guarantee that the only solution to the empty space field equations (see §2) is given by equation (1.1) giving rise to the so-called Birkhoff’s theorem [10–12]. The key point of this paper is that if these simplifying coordinate transformations are not made, another solution opportunity presents itself which suggests an improved coordinate chart to that of Schwarzschild which contains only one global singularity at the origin *r*=0 and not a coordinate singularity which is often studied in Schwarzschild geometry. Furthermore, the alternative solution allows for the generalization of the Eddington–Finkelstein and Kruskal–Szekeres coordinate transformations. By making the usual simplifying assumptions and coordinate transformations at the outset, the solution presented below is excluded.

In §2, a description of the governing equations which leads to both solutions is presented. In §3, we present a novel derivation of the Schwarzschild solution using the governing equations presented in the prior section. In §4, we derive a new solution to the empty space field equations of general relativity and corresponding generalizations of the Eddington–Finkelstein transformation and the Kruskal–Szekeres coordinates. Finally, the non-zero, independent Christoffel symbols and mixed and covariant Einstein tensor components associated with equation (1.4) are presented in appendices A–D.

## 2. Governing equations

Throughout the remainder of the present paper, attention is restricted to the space–time metric given by equation (1.4). The metric tensor *g*_*ab*_ and the inverse metric tensor (*g*_*ab*_)^−1^ are given by

2.1 $$g_{ab}=\left[\begin{array}{cccc} a & b & 0 & 0\\ b & -c & 0 & 0\\ 0 & 0 & -r^{2} & 0\\ 0 & 0 & 0 & -r^{2}\sin^{2}\theta \end{array}\right]\;\mathrm{and}\;{\left(g_{ab}\right)}^{-1}=\left[\begin{array}{cccc} \frac{c}{\omega } & \frac{b}{\omega } & 0 & 0\\ \frac{b}{\omega } & -\frac{a}{\omega } & 0 & 0\\ 0 & 0 & -r^{-2} & 0\\ 0 & 0 & 0 & -{\left(r^{2}\sin^{2}\theta \right)}^{-1} \end{array}\right],$$

where *a*,*b* and *c* are functions of both space and time and *ω*(*r*,*t*)≡*ac*+*b*^2^.

We determine the unknown functions *a*,*b* and *c* when applied to the empty space field equations of general relativity [7]

2.2 $$G_{ab}=0\;\mathrm{and}\;G_{ab}\equiv R_{ab}-\frac{1}{2}g_{ab}R,$$

where *R*_*ab*_ and *R* are the Ricci tensor and Ricci scalar, respectively (cf. [3,7]). The first two equations under consideration are equations (B 2) and (B 3) which are given by

$$\begin{array}{rl} G_{00} & =\frac{\left(a(a^{2}c_{r}-a_{r}b^{2}+2abb_{r})r+\omega^{2}-a\omega \right)}{{\left(\omega r\right)}^{2}}-\frac{b(a^{2}c_{t}+2abb_{t}-a_{t}b^{2})}{\omega^{2}r}=0\\ \;\mathrm{and}\;G_{01} & =\frac{a(\omega c_{t}+abc_{r}+a_{r}bc+2b^{2}b_{r})}{\omega^{2}r}-\frac{\left(b(abc_{t}+(aa_{r}+ba_{t}-2ab_{t})c+b^{2}a_{r})r-\omega^{2}+a\omega \right)}{{\left(\omega r\right)}^{2}}=0, \end{array}$$

respectively. After some simplification and introducing *u*(*r*,*t*)=*ra*/*ω*, we may rewrite equations (B 2) and (B 3) as a system of linear partial differential equations

2.3 $$a(r,t)=au_{r}-bu_{t}\;\mathrm{and}\;b(r,t)=bu_{r}+cu_{t},$$

where we have introduced the subscript notation to indicate partial derivatives with respect to the respective coordinate. The above system of equations can be immediately solved to give *u*_*r*_=1 and *u*_*t*_=0. We obtain an expression for *a*(*r*,*t*) by integrating *u*_*r*_=1, thus

2.4 $$a(r,t)=\omega (r,t)\left(1+\frac{r_{0}}{r}\right),$$

where *r*_0_ is introduced as a constant of integration. Next, consider equation (A 4)

$$G_{1}^{0}=\frac{\left(\omega c_{t}+b(ac_{r}+a_{r}c+2bb_{r})\right)}{\omega^{2}r}=0,$$

which, upon simplification yields

2.5 $$\omega c_{t}+b\omega_{r}=0.$$

The remaining equations of interest arise from manipulations of equations (B 4) and (B 5).

First, consider equation (B 4)

$$G_{11}=\frac{c((abc_{t}+(aa_{r}+ba_{t}-2ab_{t})c+a_{r}b^{2})r-\omega^{2}+a\omega )}{{\left(\omega r\right)}^{2}}+\frac{b(\omega c_{t}+b(ac_{r}+ca_{r}+2bb_{r}))}{\omega^{2}r}=0,$$

which, after some rearranging can be expressed as

$$\frac{u_{r}}{c}-\frac{r}{c}{\left(\frac{b}{\omega }\right)}_{t}-\frac{r}{\omega }{\left(\frac{b}{c}\right)}_{t}=\frac{1}{c},$$

by introducing expressions *v*=*rb*/*ω* and *w*=*rc*/*ω* and making use of *u*_*r*_=1, the above now reads

2.6 $$\frac{2v_{t}}{v}=\frac{w_{t}}{w}.$$

Finally, upon integration, an expression for the unknown function *b*(*r*,*t*) is obtained, given by

2.7 $$b(r,t)={\left[\frac{\omega (r,t)}{r}f(r)c(r,t)\right]}^{1/2},$$

where *f*(*r*) is obtained after integrating equation (2.6) with respect to time. By substituting equations (2.4) and (2.7) into *ω*(*r*,*t*)=*ac*+*b*^2^, we determine the unknown function *c*(*r*,*t*) in terms of *f*(*r*), which is given by

2.8 $$c(r,t)=\frac{r}{r+r_{0}+f(r)}.$$

It is clear from this equation that *c*(*r*,*t*) is at most a function of *r* only, so that *c*(*r*,*t*)=*c*(*r*). From equation (2.5), assuming that *b*(*r*,*t*) is non-zero and using the fact that *c*_*t*_=0, we can deduce that *ω*(*r*,*t*) is at most a function of time, so that *ω*(*r*,*t*)=*ω*(*t*). By substituting equation (2.8) into equation (2.7), we find

2.9 $$b(r,t)={\left[\frac{\omega (t)f(r)}{r+r_{0}+f(r)}\right]}^{1/2}.$$

Finally, by multiplying equation (B 5) by −4*ω*(*t*)^2^/*r*, the remaining equation of interest is given by

$$\begin{array}{rl} \frac{-4\omega^{2}G_{22}}{r} & =(2\omega c_{tt}-ac_{t}^{2}-(a_{t}c+a_{r}b+2bb_{t})c_{t}\\ & \;+(aa_{r}+a_{t}b-2ab_{t})c_{r}+(a_{r}^{2}-2aa_{rr}+4ab_{rt}-2a_{t}b_{r})c\\ & \;-2b(a_{r}b_{r}-a_{rr}b+2b_{r}b_{t}+2bb_{rt}))r-2a(ac_{r}+bc_{t})\\ & \;+2(2ab_{t}-a_{t}b-aa_{r})c+4b(ab_{r}-a_{r}b)=0, \end{array}$$

where, after some simplification and using *ω*=*ω*(*t*) and *c*_*t*_=0 the above equation becomes

$$4\omega {\left(rb\right)}_{rt}-2\omega_{t}{\left(rb\right)}_{r}-2ab_{t}{\left(rc\right)}_{r}-2\omega (t){\left(ra\right)}_{rr}-b\omega_{t}-2\omega b_{t}=0.$$

Dividing the above equation by 2*ω*(*t*)^3/2^, we deduce

$$2{\left(\frac{{\left(rb\right)}_{r}}{\omega {\left(t\right)}^{1/2}}\right)}_{t}-{\left(\frac{{\left(ra\right)}_{r}}{\omega {\left(t\right)}^{1/2}}\right)}_{r}+{\left(\frac{b}{\omega {\left(t\right)}^{1/2}}\right)}_{t}-ab_{t}\left(\frac{{\left(rc\right)}_{r}}{\omega {\left(t\right)}^{1/2}}\right)=0.$$

To simplify the above expressions further, consider the expression for *b*(*r*,*t*) which is given by equation (2.9). It is immediately obvious that the only time-dependent component will cancel with the denominator in both the first and third terms. Also, the second term in the above expression is a second derivative of a linear function, hence, the only non-zero term in the above equation becomes

2.10 $$\left(1+\frac{r_{0}}{r}\right)\frac{\omega_{t}(t)}{2}{\left[\frac{f(r)}{r+r_{0}+f(r)}\right]}^{1/2}{\left(rc\right)}_{r}=0.$$

Evidently, this can be satisfied in three different ways:

$$\left(i\right)\;f(r)\equiv 0,\;\left(\mathrm{ii}\right)\;\omega_{t}(t)\equiv 0\;\mathrm{and}\;\left(\mathrm{iii}\right)\;{\left(rc\right)}_{r}\equiv 0.$$

In the first two cases, Schwarzschild-like solutions are obtained which, under certain conditions, give precisely the Schwarzschild solution. More importantly, case (iii) gives rise to a new solution opportunity which leads to generalizations of the Eddington–Finkelstein transformation and the Kruskal–Szekeres coordinates.

## 3. Schwarzschild solution

The three situations in which equation (2.10) is satisfied are as follows:

*Case* (*i*): *f*(*r*)=0.

By examining equations (2.4), (2.9) and (2.8) under the condition *f*(*r*)=0, it is obvious that *b*(*r*,*t*)=0 and

3.1 $$a(r,t)=\omega (t)\left(1+\frac{r_{0}}{r}\right)\;\mathrm{and}\;c(r,t)=\frac{r}{\left(r+r_{0}\right)},$$

with *ω*(*t*)=1 producing precisely the Schwarzschild solution.

*Case* (*ii*): *ω*_*t*_(*t*)=0.

The second case arises when *ω*_*t*_(*t*)=0, or, equivalently *ω*(*t*) is given by a constant. By setting *ω*(*t*)=*α*^2^, we can write the solution in the form *ac*+*b*^2^=*α*^2^, where

3.2 $$\sqrt{ac}=\alpha \;\cos\;\psi_{0}\;\mathrm{and}\;b=\alpha \;\sin\;\psi_{0},$$

for some constant angle *ψ*_0_. Using the expressions derived for the functions *a*,*b* and *c* given by equations (2.4), (2.9) and (2.8), respectively, in conjunction with equation (3.2), we obtain an expression for *f*(*r*), namely

3.3 $$f(r)=(r+r_{0})\;\tan^{2}\psi_{0}.$$

Finally, by substituting the above into equations (2.8) and (2.9), we can show

3.4 $$a(r,t)=\alpha^{2}\left(1+\frac{r_{0}}{r}\right),\;b(r,t)=\alpha \;\sin\;\psi_{0}\;\mathrm{and}\;c(r,t)=\frac{r}{r+r_{0}}\;\cos^{2}\psi_{0}.$$

The precise Schwarzschild solution is realized by imposing the conditions *α*=1 and *ψ*_0_=0.

## 4. Derivation of new solution and generalized transformations and coordinates

The third and final case to be considered is of particular interest as it produces an alternative solution to the empty space field equations of general relativity.

*Case* (*iii*): (*rc*)_*r*_=0.

The remaining condition satisfying equation (2.10) is given by (*rc*)_*r*_=0, which, upon integration yields *c*(*r*)=*β*/*r*, where *β* is a constant of integration. Comparing this with equation (2.8) an expression for *f*(*r*) is obtained

4.1 $$f(r)=\frac{r^{2}}{\beta }-r-r_{0}.$$

By substituting the above into equation (2.7), it can be shown that

4.2 $$b(r,t)={\left[\omega (t)\left(1-\frac{\beta (r+r_{0})}{r^{2}}\right)\right]}^{1/2}.$$

The new derived expressions for the functions *a*,*b* and *c* constitute an alternative exact solution of the field equations of general relativity where the line element is

4.3 $${\left(ds\right)}^{2}=\omega (t)\left(1+\frac{r_{0}}{r}\right)dt^{2}+2{\left[\omega (t)\left(1-\frac{\beta (r+r_{0})}{r^{2}}\right)\right]}^{1/2}dr\;dt-\frac{\beta }{r}\;dr^{2}-r^{2}\;d\Omega^{2}.$$

Evidently, we may now introduce *τ*, such that d*τ*=*ω*(*t*)^1/2^ d*t*, in which case, the above equation now reads

4.4 $${\left(ds\right)}^{2}=\left(1+\frac{r_{0}}{r}\right)d\tau^{2}+2{\left[1-\frac{\beta (r+r_{0})}{r^{2}}\right]}^{1/2}dr\;d\tau -\frac{\beta }{r}\;dr^{2}-r^{2}\;d\Omega^{2}.$$

For *β*=0, the line element becomes

4.5 $${\left(ds\right)}^{2}=\left(1+\frac{r_{0}}{r}\right)d\tau^{2}+2\;dr\;d\tau -r^{2}\;d\Omega^{2}.$$

Using the computer algebra system (CAS) Maxima [13], the three line elements given by equations (4.3)–(4.5) can all be independently verified as bona fide solutions of the field equations, where *α* and *β* denote arbitrary constants and *ω*(*t*) denotes an arbitrary function of time. We observe that equation (4.5) corresponds to the outgoing Eddington–Finkelstein coordinates and since we have arbitrarily assigned the positive root for *b* in equation (4.2), we might similarly retrieve the ingoing Eddington–Finkelstein coordinates by adopting the negative sign. We further observe that both the Schwarzschild solution and equation (3.4) with *α*=1 have the common structure that can be written as

4.6 $${\left(ds\right)}^{2}=\left(1+\frac{r_{0}}{r}\right)d\tau^{2}+2\;\sin\psi \;dr\;d\tau -\frac{\cos^{2}\psi }{1+r_{0}/r}\;dr^{2}-r^{2}\;d\Omega^{2},$$

where *ψ* has the constant value *ψ*_0_ for the Schwarzschild solution given by equation (3.4) and a variable value $\sin^{-1}({\left[1-\beta (r+r_{0})/r^{2}\right]}^{1/2})$ for the solution derived here involving the new constant *β*. As inferred by the so-called Birkhoff’s theorem, the important question arises as to whether equation (4.4) is locally isometric to the Schwarzschild solution. The answer is in the affirmative and the specific details are given in §5. Although the new solution is locally isometric to the Schwarzschild solution, the authors note the new coordinate chart given by equation (4.4) avoids the coordinate singularity at *r*=*r*_0_ and allows for radially ingoing/outgoing particles to pass freely between this region which is normally associated with Eddington–Finkelstein coordinates.

The line element given by equation (4.6) can be shown to become

4.7 $${\left(ds\right)}^{2}=\left(1+\frac{r_{0}}{r}\right)(d\tau^{2}+2\;\sin\psi \;dr^{⋆}\;d\tau -\cos^{2}\psi {\left(dr^{⋆}\right)}^{2})-r^{2}\;d\Omega^{2},$$

where d*r*^⋆^ and sin⁡ψ are given by

4.8 $$dr^{⋆}=\frac{dr}{1+r_{0}/r},\;\sin\psi ={\left[1-\frac{\beta (r+r_{0})}{r^{2}}\right]}^{1/2}$$

and *ψ*=*π*/2 in the case of equation (4.5). By performing the transformation *τ*=*τ*^⋆^+*ρ*(*r*^⋆^), it is clear that equation (4.7) becomes

4.9 $${\left(ds\right)}^{2}=\left(1+\frac{r_{0}}{r}\right){\left((d\tau^{⋆}\right)}^{2}+2(\sin\psi +\rho^{\prime }(r^{⋆}))\;d\tau^{⋆}\;dr^{⋆}+[{\left(\sin\psi +\rho^{\prime }(r^{⋆})\right)}^{2}-1]{\left(dr^{⋆}\right)}^{2})-r^{2}\;d\Omega^{2},$$

where the prime denotes differentiation with respect to *r*^⋆^. From the structure of equation (4.9), it is apparent that *ρ*(*r*^⋆^) can be chosen to produce any desired equation of the form of equation (4.7). Thus, as an example we may obtain the Schwarzschild line element by making

4.10 $$\frac{d\rho }{dr^{⋆}}+\sin\;\psi =\sin\;\psi_{0},$$

from which we may deduce that the function *ρ*(*r*^⋆^) is determined by performing the integration

4.11 $$d\rho =(r\;\sin\;\psi_{0}-{\left[r^{2}-\beta (r+r_{0})\right]}^{1/2})\frac{dr}{r+r_{0}},$$

which clearly admits a range of analytical expressions depending upon the value of *β*, noting the greatly simplified form arising from the special case *β*=−4*r*_0_.

As an illustration of the above, we derive the unknown function *ρ*(*r*^⋆^) which allows for the coordinate transformation from Schwarzschild to Eddington–Finkelstein coordinates. In Schwarzschild geometry *ψ*_0_=*β*=0 and hence, equation (4.11) becomes

4.12 $$\rho (r)=\pm \int \frac{r}{r+r_{0}}\;dr=\pm (r-r_{0}\;\ln\;(r+r_{0})+C),$$

where *C* is a constant of integration. Substituting equation (4.12) into *τ*=*τ*^⋆^+*ρ*(*r*) and applying to the Schwarzschild line element gives precisely the outgoing and ingoing Eddington–Finkelstein coordinates depending on the choice of sign. We note that *τ*=*τ*^⋆^+*ρ*(*r*) together with equation (4.11) provides a generalization of the Eddington–Finkelstein transformation.

To extend the Kruskal–Szekeres coordinate transformation, we define the variables *ξ* and *η* through the differential relations

4.13 $$d\xi =dr^{⋆}+d\tau +\sin\psi \;dr^{⋆}\;\mathrm{and}\;d\eta =dr^{⋆}-d\tau -\sin\psi \;dr^{⋆},$$

where *r*^⋆^, *τ* and sin⁡ψ are all as given above. Explicitly, *ξ* and *η* are given by (derivation given in appendix D)

4.14 $$\begin{array}{rl} \xi & =\tau +\int \frac{r}{r+r_{0}}\left(1+{\left[1-\frac{\beta (r+r_{0})}{r^{2}}\right]}^{1/2}\right)\;dr,\\ & =\tau +r+{\left[r^{2}-\beta (r+r_{0})\right]}^{1/2}-\left(r_{0}+\frac{\beta }{2}\right)\;\ln\;\left({\left[r^{2}-\beta (r+r_{0})\right]}^{1/2}+r-\frac{\beta }{2}\right)\\ & \;-r_{0}\;\ln\;\left({\left[r^{2}-\beta (r+r_{0})\right]}^{1/2}-r-\frac{\beta (r+r_{0})}{2r_{0}}\right)+\xi_{0}\\ \;\mathrm{and}\;\eta & =-\tau +\int \frac{r}{r+r_{0}}\left(1-{\left[1-\frac{\beta (r+r_{0})}{r^{2}}\right]}^{1/2}\right)\;dr,\\ & =-\tau +r-2r_{0}\;\ln\;(r+r_{0})-{\left[r^{2}-\beta (r+r_{0})\right]}^{1/2}\\ & \;+\left(r_{0}+\frac{\beta }{2}\right)\;\ln\;\left({\left[r^{2}-\beta (r+r_{0})\right]}^{1/2}+r-\frac{\beta }{2}\right)\\ & \;+r_{0}\;\ln\;\left({\left[r^{2}-\beta (r+r_{0})\right]}^{1/2}-r-\frac{\beta (r+r_{0})}{2r_{0}}\right)+\eta_{0}, \end{array}\}$$

where *ξ*_0_ and *η*_0_ are introduced as arbitrary constants of integration, noting that *ξ* and *η* are essentially *r*^⋆^+*τ*^⋆^ and *r*^⋆^−*τ*^⋆^, respectively, where *τ*^⋆^ is precisely as defined in the previous section. In the above integral evaluations, it is assumed that the arguments of all logarithms are positive; in other cases, slightly modified formulae may apply. On evaluating the product *dξ* *dη*, we see that equation (4.7) becomes

4.15 $${\left(ds\right)}^{2}=-\left(1+\frac{r_{0}}{r}\right)d\xi \;d\eta -r^{2}\;d\Omega^{2},$$

where we can identify the product *dξ* *dη* as the generalized ingoing/outgoing Eddington–Finkelstein coordinates. Let us propose generalized coordinates R and T such that

4.16 $$R+T=e^{-\xi /2r_{0}}\;\mathrm{and}\;R-T=e^{-\eta /2r_{0}},$$

as the new extended Kruskal–Szekeres coordinates for *β*≠0, where *ξ* and *η* are defined as in equation (4.13) since when *ψ*=0 we have

4.17 $$\xi =\tau +r-r_{0}\;\ln\;(r+r_{0})+\xi_{0},\;\eta =-\tau +r-r_{0}\;\ln\;(r+r_{0})+\eta_{0}$$

and on adopting the values $\xi_{0}=\eta_{0}=r_{0}\;\ln\;(r_{0})$, we may deduce the relations for the standard Kruskal–Szekeres coordinates

4.18 $$\begin{array}{rl} R+T & ={\left(1+\frac{r_{0}}{r}\right)}^{1/2}e^{-r/2r_{0}}e^{-\tau /2r_{0}},\\ R-T & ={\left(1+\frac{r_{0}}{r}\right)}^{1/2}e^{-r/2r_{0}}e^{\tau /2r_{0}} \end{array}\}$$

and, therefore,

4.19 $$\begin{array}{rl} R & ={\left(1+\frac{r_{0}}{r}\right)}^{1/2}e^{-r/2r_{0}}\cosh\left(\frac{\tau }{2r_{0}}\right)\\ \;\mathrm{and}\;T & =-{\left(1+\frac{r_{0}}{r}\right)}^{1/2}e^{-r/2r_{0}}\sinh\left(\frac{\tau }{2r_{0}}\right), \end{array}\}$$

where *R* and *T* denote standard Kruskal–Szekeres coordinates. Finally, the line element given by equation (4.15) in generalized Kruskal–Szekeres coordinates is given by

4.20 $${\left(ds\right)}^{2}=4\frac{r_{0}^{3}}{r}e^{r/r_{0}}(dT^{2}-dR^{2})-r^{2}\;d\Omega^{2}.$$

We note that, in general, the line element given by equation (4.20) applies for all *β* and is obvious from the relation

4.21 $$\xi +\eta =2r-2r_{0}\;\ln\;\left(1+\frac{r}{r_{0}}\right).$$

Thus, for all *β*, we have with the above definition

4.22 $$R^{2}-T^{2}=\left(1+\frac{r_{0}}{r}\right)e^{-r/r_{0}},$$

assuming we adopt the same values for the arbitrary constants *ξ*_0_ and *η*_0_.

## 5. Conclusion

We have derived a spherically symmetric, static solution of the empty space field equations of general relativity, where the line element given by equation (4.4) involves two arbitrary constants. By consulting the weak field limit [3], we can immediately identify *r*_0_=−2*GM*/*c*^2^ as the Schwarzschild radius. The second arbitrary constant allows for the generalizations of the well-known Eddington–Finkelstein transformation and the Kruskal–Szekeres coordinates. Furthermore, a variety of analytical forms for the extended Eddington–Finkelstein transformation and the Kruskal–Szekeres coordinates are derivable depending on the value of *β*, and one derivation is provided in appendix D. The key point is that this solution does not arise if the usual assumptions leading to the Schwarzschild solution are made at the outset. We note the Schwarzschild solution is formally obtained by introducing a new time variable *u* in equation (4.4) through the differential relation given by

5.1 $$du=d\tau +\frac{{\left[1-\beta (r+r_{0})/r^{2}\right]}^{1/2}}{1+r_{0}/r}\;dr.$$

Finally, the authors note that this approach can be easily generalized to the Reissner–Nordstrom solution in GR [3].

## Data accessibility

This article has no additional data.

## Appendix A. Non-zero mixed Einstein tensor

The components of the mixed Einstein tensor are obtained using

A 1 $$G_{b}^{a}=R_{b}^{a}-\frac{1}{2}\delta_{b}^{a}R,$$

where $\delta_{b}^{a}$, $R_{b}^{a}$ and *R* denote the Kronecker delta, the Ricci tensor and the Ricci scalar, respectively. On application to equation (1.4), the non-zero, independent components become

A 2 $$\begin{array}{r} G_{0}^{0}=\frac{\left((a^{2}c_{r}-a_{r}b^{2}+2abb_{r})r+\omega^{2}-a\omega \right)}{{\left(\omega r\right)}^{2}}, \end{array}$$

A 3 $$\begin{array}{r} G_{0}^{1}=-\frac{\left(a^{2}c_{t}+2abb_{t}-a_{t}b^{2}\right)}{\omega^{2}r}, \end{array}$$

A 4 $$\begin{array}{r} G_{1}^{0}=\frac{\left(\omega c_{t}+(ac_{r}+a_{r}c)b+2b^{2}b_{r}\right)}{\omega^{2}r}, \end{array}$$

A 5 $$\begin{array}{r} G_{1}^{1}=-\frac{\left((abc_{t}+(a_{t}b+aa_{r}-2ab_{t})c+a_{r}b^{2})r-\omega^{2}+a\omega \right)}{{\left(\omega r\right)}^{2}} \end{array}$$

A 6 $$\begin{array}{r} \;\mathrm{and}\;G_{2}^{2}=\frac{\begin{array}{c} ((2\omega c_{tt}-ac_{t}^{2}-(a_{t}c+a_{r}b+2bb_{t})c_{t}\\ \;+(aa_{r}+a_{t}b-2ab_{t})c_{r}+(a_{r}^{2}-2aa_{rr}+4ab_{rt}-2a_{t}b_{r})c\\ \;-2b(a_{r}b_{r}-a_{rr}b+2b_{r}b_{t}+2bb_{rt}))r-2a(ac_{r}+bc_{t})\\ \;+2(2ab_{t}-a_{t}b-aa_{r})c+4b(ab_{r}-a_{r}b)) \end{array}}{4\omega^{2}r}. \end{array}$$

## Appendix B. Non-zero covariant Einstein tensor

The components of the covariant Einstein tensor are determined from

B 1 $$G_{ab}=R_{ab}-\frac{1}{2}g_{ab}R$$

and when applied to equation (1.4), the non-zero, independent components are given by

B 2 $$\begin{array}{rl} G_{00} & =\frac{\left(a(a^{2}c_{r}-a_{r}b^{2}+2abb_{r})r+\omega^{2}-a\omega \right)}{{\left(\omega r\right)}^{2}}-\frac{b(a^{2}c_{t}+2abb_{t}-a_{t}b^{2})}{\omega^{2}r}, \end{array}$$

B 3 $$\begin{array}{r} G_{01}=\frac{a(\omega c_{t}+abc_{r}+a_{r}bc+2b^{2}b_{r})}{\omega^{2}r}-\frac{\left(b(abc_{t}+(aa_{r}+ba_{t}-2ab_{t})c+b^{2}a_{r})r-\omega^{2}+a\omega \right)}{{\left(\omega r\right)}^{2}}, \end{array}$$

B 4 $$\begin{array}{r} G_{11}=\frac{c((abc_{t}+(aa_{r}+ba_{t}-2ab_{t})c+a_{r}b^{2})r-\omega^{2}+a\omega )}{{\left(\omega r\right)}^{2}}+\frac{b(\omega c_{t}+abc_{r}+bca_{r}+2b^{2}ba_{r})}{\omega^{2}r} \end{array}$$

B 5 $$\begin{array}{r} \;\mathrm{and}\;G_{22}=-\frac{\begin{array}{c} r((2\omega c_{tt}-ac_{t}^{2}-(a_{t}c+a_{r}b+2bb_{t})c_{t}\\ +(aa_{r}+a_{t}b-2ab_{t})c_{r}+(a_{r}^{2}-2aa_{rr}+4ab_{rt}-2a_{t}b_{r})c\\ -2b(a_{r}b_{r}-a_{rr}b+2b_{r}b_{t}+2bb_{rt}))r-2a(ac_{r}+bc_{t})\\ +2(2ab_{t}-a_{t}b-aa_{r})c+4b(ab_{r}-a_{r}b)) \end{array}}{4\omega^{2}}. \end{array}$$

## Appendix C. Non-zero Christoffel symbols of the second kind

The Christoffel symbols of the second kind are given by

C 1 $$\Gamma_{bc}^{a}=\frac{g^{ad}(\partial_{c}g_{bd}+\partial_{b}g_{cd}-\partial_{d}g_{bc})}{2},$$

where ∂_*c*_ denotes ∂/∂*x*^*c*^ and when applied to the metric given by equation (1.4) the non-zero, independent Christoffel symbols become

C 2 $$\begin{array}{rl} \Gamma_{00}^{0} & =\frac{\left(a_{t}c-a_{r}b+2bb_{t}\right)}{2\omega },\;\Gamma_{01}^{0}=\frac{\left(a_{r}c-bc_{t}\right)}{2\omega },\\ \Gamma_{11}^{0} & =\frac{\left(cc_{t}-bc_{r}+2b_{r}c\right)}{2\omega },\;\Gamma_{22}^{0}=\frac{br}{\omega },\\ \Gamma_{33}^{0} & =\frac{\left(br\sin^{2}\theta \right)}{\omega },\;\Gamma_{00}^{1}=\frac{a_{t}b+aa_{r}-2ab_{t}}{2\omega },\\ \Gamma_{01}^{1} & =\frac{\left(ac_{t}+a_{r}c\right)}{2\omega },\;\Gamma_{11}^{1}=\frac{bc_{t}+ac_{r}+2bb_{r}}{2\omega },\\ \Gamma_{22}^{1} & =-\frac{ar}{\omega },\;\Gamma_{33}^{1}=-\frac{\left(ar\sin^{2}\theta \right)}{\omega },\\ \Gamma_{12}^{2} & =\frac{1}{r},\;\Gamma_{33}^{2}=-\cos\theta \sin\theta \\ \;\mathrm{and}\;\Gamma_{13}^{3} & =\frac{1}{r},\;\Gamma_{23}^{3}=\cot\theta . \end{array}$$

## Appendix D. Integration formulae

To explicitly determine the expression for *ξ* from equation (4.14) consider the expanded expression given by

D 1 $$\xi =\tau +\int \frac{r}{r+r_{0}}\;dr+\int \frac{{\left[r^{2}-\beta (r+r_{0})\right]}^{1/2}}{r+r_{0}}\;dr,$$

where attention will be restricted to the second integral. We begin by making the substitution *x*=(*r*+*r*_0_)^−1^, where d*r*=−*dx*/*x*^2^ so that the second integral in equation (D.14) becomes

D 2 $$-\int {\left[{\left(xr_{0}\right)}^{2}-x(2r_{0}+\beta )+1\right]}^{1/2}\frac{dx}{x^{2}}.$$

The integral given by equation (D.15) becomes

D 3 $$\frac{\sqrt{R}}{x}+\left(r_{0}+\frac{\beta }{2}\right)\int \frac{dx}{x\sqrt{R}}-r_{0}^{2}\int \frac{dx}{\sqrt{R}},$$

where *R*=(*r*_0_*x*)^2^−(2*r*_0_+*β*)*x*+1 and to evaluate we consult [14], pp. 81, 84 from which we may deduce

D 4 $$\begin{array}{rl} \int \frac{dx}{x\sqrt{R}} & =-\;\ln\;\left[\frac{2-(2r_{0}+\beta )x+2\sqrt{R}}{x}\right]\\ \;\mathrm{and}\;\int \frac{dx}{\sqrt{R}} & =\frac{1}{r_{0}}\;\ln\;[2r_{0}\sqrt{R}+2r_{0}^{2}x-(2r_{0}+\beta )], \end{array}\}$$

respectively. Furthermore, using the relation

D 5 $$\sqrt{R}=\frac{{\left[r^{2}-\beta (r+r_{0})\right]}^{1/2}}{r+r_{0}},$$

with equation (D.17), we may evaluate equation (D.16) completely given by

D 6 $$\begin{array}{rl} \xi & =\tau +r+{\left[r^{2}-\beta (r+r_{0})\right]}^{1/2}-\left(r_{0}+\frac{\beta }{2}\right)\;\ln\;\left({\left[r^{2}-\beta (r+r_{0})\right]}^{1/2}+r-\frac{\beta }{2}\right)\\ & \;-r_{0}\;\ln\;\left({\left[r^{2}-\beta (r+r_{0})\right]}^{1/2}-r-\frac{\beta (r+r_{0})}{2r_{0}}\right)+\xi_{0} \end{array}$$

and the expression for *η* may be similarly obtained.
